# Modelling Transcapillary Transport of Fluid and Proteins in Hemodialysis Patients

**DOI:** 10.1371/journal.pone.0159748

**Published:** 2016-08-02

**Authors:** Mauro Pietribiasi, Jacek Waniewski, Alicja Załuska, Wojciech Załuska, Bengt Lindholm

**Affiliations:** 1 Institute of Biocybernetics and Biomedical Engineering, Warsaw, Poland; 2 Department of Rehabilitation and Physiotherapy, Medical University of Lublin, Lublin, Poland; 3 Department of Nephrology, Medical University of Lublin, Lublin, Poland; 4 Baxter Novum and Renal Medicine, Karolinska Institutet, Stockholm, Sweden; Emory University, UNITED STATES

## Abstract

**Background:**

The kinetics of protein transport to and from the vascular compartment play a major role in the determination of fluid balance and plasma refilling during hemodialysis (HD) sessions. In this study we propose a whole-body mathematical model describing water and protein shifts across the capillary membrane during HD and compare its output to clinical data while evaluating the impact of choosing specific values for selected parameters.

**Methods:**

The model follows a two-compartment structure (vascular and interstitial space) and is based on balance equations of protein mass and water volume in each compartment. The capillary membrane was described according to the three-pore theory. Two transport parameters, the fractional contribution of large pores (α_LP_) and the total hydraulic conductivity (LpS) of the capillary membrane, were estimated from patient data. Changes in the intensity and direction of individual fluid and solute flows through each part of the transport system were analyzed in relation to the choice of different values of small pores radius and fractional conductivity, lymphatic sensitivity to hydraulic pressure, and steady-state interstitial-to-plasma protein concentration ratio.

**Results:**

The estimated values of LpS and α_LP_ were respectively 10.0 ± 8.4 mL/min/mmHg (mean ± standard deviation) and 0.062 ± 0.041. The model was able to predict with good accuracy the profiles of plasma volume and serum total protein concentration in most of the patients (average root-mean-square deviation < 2% of the measured value).

**Conclusions:**

The applied model provides a mechanistic interpretation of fluid transport processes induced by ultrafiltration during HD, using a minimum of tuned parameters and assumptions. The simulated values of individual flows through each kind of pore and lymphatic absorption rate yielded by the model may suggest answers to unsolved questions on the relative impact of these not-measurable quantities on total vascular refilling and fluid balance.

## Introduction

Every year 600000 patients, in Europe and USA together, are treated for end-stage renal diseases (ESRD) with hemodialysis therapy (HD) [1]. Even with the many improvements received in the course of the past decades, which allowed HD to become a widespread routine procedure, there are still many complications that impose a burden on the patient organism. Intradialytic hypotensive (IDH) episodes are one of the most common complications, occurring in at least 20% of the treatments [2–4]. Although it is widely documented that IDH events are mainly related to the application of a high rate of ultrafiltration during the HD session, the exact mechanism(s) leading to the hypotensive collapse is still under debate, as are the best methods to prevent it.

Almost certainly one of the recognized triggers of IDH is the presence of too rapid water removal coupled with an inadequate refilling of the vascular space. Vascular refilling is driven by changes in the equilibrium of the Starling forces and the action of the lymphatic system. Because most of these factors are difficult to measure in a non-invasive way, mathematical models have been widely used to explain the mechanisms determining the efficacy and efficiency of vascular refilling, and may help in studying the reasons behind IDH [5–10].

Agreement can be found in literature on the prominent importance of the kinetics of plasma proteins in the regulation of refilling flow; while the capillary wall is almost perfectly permeable to the passage of small (neutral and ionic) solutes, the oncotic pressure exerted by the different protein concentration in plasma and interstitium plays a major role in determining the shifts of fluid [11]. Another parameter identified as highly influential for the performance of the refilling processes is the filtration coefficient (capillary surface area times hydraulic conductance) of the capillary walls, again a quantity we have no means to measure directly, although in spite of that it has been often the subject of investigation [9, 10, 12–15].

Although some new methods of modelling the refilling process and the estimation of the parameters involved have been tried in the course of the years, most of the models proposed were based on fairly old publications with little deviations from a model structure that was demonstrated to be simple yet robust, offering good results in its various iterations [10, 16, 17]. Some problems, as the role and variability of lymphatic flow in the refilling process during the HD session, the changes in interstitial fluid pressure with the change in fluid overload, and the heteroporous structure of the capillary wall, have not yet received extensive attention.

In this study we propose a two-compartment model of water transport and serum protein kinetics, and validate it using clinical data collected in a cohort of HD patients to obtain an estimation of the components of the refilling flow during the water removal. We apply the model on all three sessions of a standard weekly cycle of HD and study how it simulates the different characteristics of the patients before each of the three sessions of the week. The flows of fluid and proteins between body compartments are analyzed assuming different values of parameters to understand how the model describes the mechanisms of refilling and proteins turnover.

Although similar models were applied previously for this purpose, our model includes more detailed descriptions of the physiological systems that control water content in different body compartments, as volume–hydraulic pressure–lymphatic flow relationships for interstitial fluid [18]. The estimation of transport parameters, achieved through comparison with real clinical hemodialysis data, allowed us to describe individual transport systems, instead of theoretical computer simulations based on the literature data (values of parameters). For the description of the capillary wall as the transport barrier, the 3-pore model that was previously shown to describe correctly wealth of experimental data was applied [19]. Those parameters whose values were decided *a priori* and not known with sufficient confidence, were varied within a physiologically reasonable range, and the results compared with the data.

The objective of this study was to compare the components of the refilling process, as simulated by the mathematical model, between different sessions of a clinical weekly HD treatment cycle, characterized by different initial fluid status of the patients. The second objective was to compare the effect, on the output of the model, of the variation of those parameters that, by necessity, are to be chosen *a priori* during the implementation.

## Methods

### Clinical data

The model proposed in this study was applied to data collected in ESRD patients during a weekly cycle of standard clinical hemodialysis. The treatment schedule comprised three HD sessions (HD1, HD2, HD3) with pre-dialytic interval of 3 days prior to the first session, and 2 days prior to the two remaining sessions.

Twenty three patients were included, 8 males and 15 females, with median age of 66 years, ranging from 38 to 84 years. The median time on dialysis was 1 year, with range 1 to 32 months (Table 1). Six patients had diabetes. Written informed consent was obtained from each patient and the study was approved by the Ethical Committee of the Lublin Medical University, Lublin, Poland.

**Table 1:** Information on the samples of patients. Age and vintage values are shown as median (range). No statistical differences were found in the parameters described.

	HD1	HD2	HD3
*Number of valid cases*	20	22	21
*% Males*	35.0	36.4	38.1
*Age (years)*	65.5 (38–84)	67 (38–84)]	65 (38–84)
*HD vintage (months)*	12 (1–32)	12 (1–32)	12 (1–32)

During the analysis of the patients’ data, some data in different sessions for three patients in HD1, one patient in HD2, and two patients in HD3 were found to have artifacts that made them unable to be used to validate our model, leading to an uneven number of cases analyzed in each session. This caused minor differences in the patients characteristics for each session; however, all sessions had in common almost all patients (difference of 1–2 subjects), and were basically representative of the same group, as shown in Table 1. Each session kept the same number of diabetic patients.

Fluid overload, normo-hydrated body weight (difference between body weight and fluid overload), and intra- and extra-cellular fluid volumes were estimated by bioimpedance spectroscopy with Body Composition Monitor (BCM, Fresenius Medical Care, Bad Homburg, Germany). Total serum protein concentration (*C*_*p*_) was measured from blood samples collected before and after the session, and at the beginning of every hour during HD. Plasma oncotic pressure was calculated from these samples using the Landis-Pappenheimer formula [20]. Fresenius CritLine (Fresenius Medical Care, Bad Homburg, Germany) was used to estimate online blood hematocrit and relative blood volume changes during water removal, which were combined to obtain changes in plasma volume (*V*_*p*_).

Plasma volume at the end of HD was calculated with an anthropometric formula [21] and extrapolated back to obtain the initial value. The volume of interstitial fluid at the end of dialysis was calculated as the difference of extracellular fluid volume and plasma volume at that time. Pre-HD values of interstitial fluid volume were recalculated from its final value plus the difference between total water removed (calculated as the difference in pre- and post-dialytic body weight) and change in plasma volume.

The dialysis settings of the three sessions are shown in Table 2. Dialysate flow was constant for all sessions at 500 mL/min.

**Table 2:** Operative conditions of the HD treatment before each session. * p-value < 0.05 when compared to the other groups.

	HD1	HD2	HD3
*Duration (min)*	238.9 ± 11.8	237.7 ± 14.8	240.7 ± 12.6
*Ultrafiltration (mL/min)*	11.7 ± 3.1*	8.6 ± 3.4	8.8 ± 2.8
*Blood flow (mL/min)*	274.2 ± 52.5	275.8 ± 51.1	274.6 ± 50.7

All the values are shown in median [quartiles] to account for the presence of outliers and the non-normality of many variables.

### Description of the model

The mathematical model here presented describes, on a whole-body level, the shifts of blood water and proteins, which occur during a hemodialysis session. The description of both fluid and proteins includes equations for two compartments, vascular and interstitial. The intracellular compartment was not considered in this implementation because intracellular fluid didn’t change much during HD, as indicated by the data (Table 3), and because it does not directly participate in the transport of serum proteins which, for simplicity, are here homogenously described as having the dimension of albumin and uniform spherical shape (Table 4) [22, 23].

**Table 3:** Characteristics of the patients measured before each HD session expressed as median [quartiles].

	HD1	HD2	HD3
*Body weight (kg)*	67.3 [57.4, 80.2]*	69.0 [57.6, 79.0]	67.9 [57.6, 78.8]
*NHBW (kg)*	65.1 [52.8, 76.2]	67.2 [54.2, 77.0]	65.7 [54.0, 76.6]
*ECV (L)*	16.3 [14.4, 19.3]*	16.0 [13.4, 17.6]	15.5 [13.4, 17.5]
*ECV drop (L)*	2.5 [2.3 3.1]*	1.8 [1.4, 2.3]	1.9 [1.4, 2.6]
*ICV (L)*	14.5 [12.1, 17.0]	15.6 [12.2, 16.7]	15.0 [11.8, 17.9]
*ICV drop (L)*	-0.4 [-0.7, -0.1]	-0.4 [-1.1, 0.1] ^a^	-0.1 [-0.4, 0.3] ^a^
*Plasma volume (L)*	3.2 [2.8, 3.4]*	3.0 [2.8, 3.3]	3.0 [2.6, 3.3]
*Fluid overload (L)*	3.0 [2.0, 3.7]*	2.2 [1.6, 2.7]	2.1 [1.1, 2.6]
*MAP (mmHg)*	93.0 [79.9, 110.5]	90.7 [78.0, 96.7]	90.3 [80.7, 99.0]
*Hematocrit (%)*	31.5 [29.2, 32.8]^+^	31.2 [29.6, 33.6]^+^	32.1 [29.8, 33.6]
*TP (g/dL)*	6.5 [6.2, 6.7]^++^	6.7 [6.4, 6.8]	6.6 [6.4, 6.8]

NHBW = normo-hydrated body weight, ECV = extracellular volume, ICV = intracellular volume, MAP = mean arterial pressure; TP = serum total protein concentration;

* p-value < 0.001 when compared to HD2 and HD3.

^+^ p = 0.04 HD1 vs. HD2;

^++^ p = 0.02 HD1 vs. HD2 and HD3;

^a^ p = 0.02 HD2 vs. HD3.

**Table 4:** Characteristics of the implemented model.

Parameter	Symbol	Value	Source
*Large pores radius*	*r*_*LP*_	250 Å	[23]
*Small pores radius*	*r*_*SP*_	45 Å	[23]
*Ultrasmall pores radius*	*r*_*UP*_	2 Å	[23]
*Albumin radius*	*r*_*alb*_	35.5 Å	[23]
*Small pores fraction of total hydraulic conductivity*	*α*_*SP*_	0.6	[23]
*Large pores reflection coefficient*	*σ*_*LP*_	0.090	Calculated
*Small pores reflection coefficient*	*σ*_*SP*_	0.974	Calculated
*Initial interstitial-to-plasma protein concentration ratio*	*R*_*0*_	0.4	Assumed a priori

**Fig 1:**
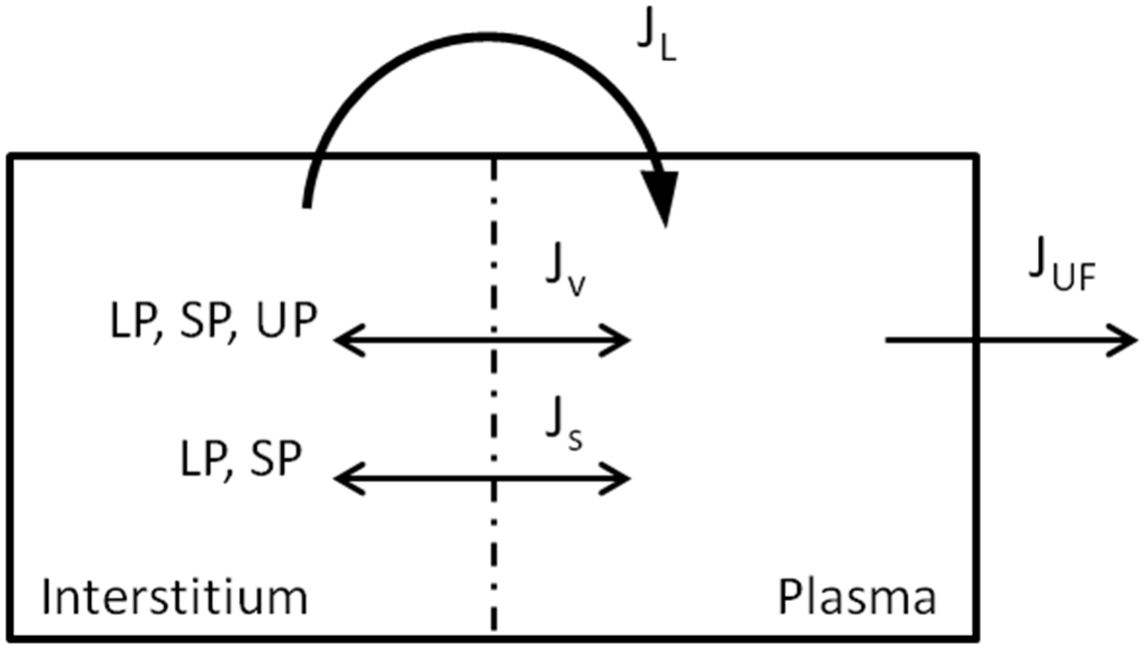
General structure of the two-compartments model. Plasma and interstitial compartments are separated by the porous capillary membrane, in which 3 types of pores are present, LP, SP, UP (large, small and ultrasmall pores). Bi-directional transport of fluid and proteins happens across the membrane through the pore system (but UP are not permeable to proteins), and is complemented by a reabsorption of fluid and solute through the lymphatic vessels. Water is removed from vascular compartment by the HD machine.

The general structure of the model is described in Fig 1. The barrier dividing the two compartments was described using the three-pore model theory [19]. Passage of both fluid and particles was assumed to take place across cylindrical, uniformly shaped and distributed pores in the capillary wall, belonging to one of three categories: large pores (LP), through which both fluid and solute (in this case albumin) can be transported; small pores (SP), of size comparable to the albumin’s sieving effect; ultrasmall pores (UP), which completely deny the passage of solutes. The radii of the membrane pores and other assumed parameters used in this model are reported in Table 4 (reflection coefficients were calculated according to [24]). To account for the presence of the water molecules shell that is formed in an aqueous solution around charged particles, a correction was introduced increasing the radius of albumin and decreasing the radii of small and large pores by 1.5 Å [23, 25]. The fractional contribution of small pores to the total hydraulic permeability of the membrane was set to 60% [26, 27], while the percentage for large pores (usually 5–10%) was estimated for each patient from the clinical data. The contribution of ultrasmall pores was calculated as:

$$\alpha_{UP}=1-\alpha_{LP}-\alpha_{SP}$$ (1)

The changes in plasma volume depend on the difference between the rate of water removal by ultrafiltration and water refilling from the interstitial space, at any given time:

$$\frac{dV_{p}}{dt}=Jv_{REF}-Jv_{UF}$$ (2)

The total refilling flow is equal to the sum of the flows of water through each type of pore, plus the contribution of lymphatics:

$$J_{REF}=Jv_{LP}+Jv_{SP}+Jv_{UP}+Jv_{L}$$ (3)

For each pore type, the water transport is expressed by the Starling equation (the subscript ‘_*X*_*’* stands for LP, SP or UP):

$$Jv_{X}=LpS\cdot \alpha_{X}\cdot [P_{i}-P_{c}-\sigma_{X}(\Pi_{i}-\Pi_{p})]$$ (4)

where *P*_*i*_, *P*_*c*_, *Π*_*i*_ and *Π*_*c*_ are the Starling forces: interstitial hydraulic pressure, capillary hydraulic pressure, interstitial oncotic pressure and capillary oncotic pressure, respectively; *α* and *σ* are, for each pore type, the fraction of the hydraulic conductance accounted for by pore-type *x* and the reflection coefficient for albumin [23, 28]. *LpS* is the capillary filtration coefficient, expressing the total hydraulic permeability of the capillary wall to water, to which each type of pore contributes.

Lymphatic flow is a function of interstitial hydraulic pressure, given by:

$$Jv_{L}(t)=Jv_{L,0}+\beta \cdot [P_{i}(t)-P_{i}(0)]$$ (5)

*Jv*_*L*,*0*_ is the value of lymphatic flow at steady state and it’s equal and opposite to the sum of the pore flows, in order to obtain a zero net balance of fluid flows at steady state; *β* is expressed by:

$$\beta =\;LS\cdot Jv_{L,0},$$

where *LS* is the lymphatic sensitivity of the tissue to increase in interstitial fluid pressure, and its value in the skeletal muscle was set to 0.4 [29]. Such value was used as a surrogate for the whole-body, given that lymphatic capillaries are contained mostly in the muscular tissue [30]. *Jv*_*L*_ was considered to be strictly a refilling flow and, for decreasing *P*_*i*_*(t)*, its minimum possible value was set to 0.

The initial volume of interstitial fluid was calculated as the difference of the measured content of extracellular water and plasma. Given the absence of the intracellular compartment from this model, it is assumed that all changes of interstitial volume are caused only by the fluid exchange with the vascular compartment, hence:

$$\frac{dV_{i}}{dt}=-Jv_{REF}$$ (6)

Where *Jv*_*REF*_ is defined by Eq 3.

The kinetics of proteins in both model compartments is defined by the sum of diffusive transport across the capillary wall and convective flows (through the pores and the lymphatic system). In the vascular space, changes in the total mass of proteins (*M*_*p*_) are expressed by the following:

$$\frac{dM_{p}}{dt}=\frac{d(C_{p}\cdot V_{p})}{dt}=Js_{DIFF}+Js_{LP}+Js_{SP}+Js_{L}$$ (7)

Because the size of UP effectively prevents all passage of big solutes through them, ultrasmall pores do not take part in proteins transport.

The diffusive term is proportional to the difference between plasma (*C*_*p*_) and interstitial (*C*_*i*_) concentrations:

$$Js_{DIFF}=PS_{T}(C_{i}-C_{p})$$ (8)

where *PS*_*T*_ *= PS*_*LP*_ *+ PS*_*SP*_ is the total diffusivity coefficient for albumin across the capillary wall. The individual contributions of each pore type involved are calculated from pore and solute radii (and from the capillary filtration coefficient) [23].

Proteins are transported in the lymphatic circulation by convection only, depending on the interstitial concentration:

$$Js_{L}=Jv_{L}\cdot C_{i}$$ (9)

For each kind of pore the convective transport of proteins is expressed by:

$$Js_{X}=(1-\sigma_{X})Jv_{X}\cdot Cm_{X}$$ (10)

where *S*_*x*_ *= (1−σ*_*x*_*)* is called the sieving coefficient for protein (albumin). *Cm*_*x*_ is the average protein concentration throughout the length of the pore, and it is calculated from pore fluid flows and the two compartments’ protein concentrations, according to the simplified Kedem-Katchalsky model [31, 32].

The change of protein mass in the interstitial space is equal and opposite to the change in plasma:

$$\frac{dM_{i}}{dt}=\frac{d(C_{i}\cdot V_{i})}{dt}=-(Js_{DIFF}+Js_{LP}+Js_{SP}+Js_{L})$$ (11)

The total protein concentration in the interstitial compartment before the start of HD (*C*_*i*,*0*_) was calculated as:

$$C_{i,0}=R_{0}\cdot C_{p,0}$$ (12)

where *R*_*0*_ was set to a fixed value for all patients (Table 4).

Both hydraulic pressures appearing in the Starling equation cannot be measured with standard clinical tools, so they had to be obtained from other sources. Interstitial pressure was calculated internally by the model from the simulated values of interstitial fluid volume, according to the empirical formula proposed in [33]:

$$P_{i}=-7.9+0.8\cdot V_{i}-0.009\cdot V_{i}{}^{2}$$ (13)

Pre-HD capillary hydraulic pressure (*P*_*c*,*0*_) was considered constant during the whole treatment [34] and was calculated by the model as follows: Let us consider the Starling equation for global water flow across the capillary, calculated at a time before the start of water removal, in an hypothetical case in which the Starling forces are perfectly balanced out:

$$0=P_{i,0}-P_{c,0}-\sigma_{T}(\Pi_{i,0}-\Pi_{c,0})$$ (14)

From this equation one can express, as a function of the other Starling forces, the value of *P*_*c*,*0*_, for which the net filtration flow at steady-state is zero. However it is known from as early as in studies by Guyton [35, 36] that in physiological conditions there is always a small net filtration of fluid, even at equilibrium, which is compensated by an equivalent lymphatic absorption. To achieve this, *P*_*c*,*0*_ should be slightly higher than the sum of the other forces, by an amount that we called *NFD*_*0*_ (net filtration drive):

$$P_{c,0}=P_{i,0}+\sigma_{T}(\Pi_{p,0}-\Pi_{i,0})+NFD_{0}$$ (15)

Rather than assuming a value *a priori*, it was decided to let *NFD*_*0*_ change to balance out the steady-state equation for protein flows:

$$Js_{DIFF,0}+Js_{LP,0}+Js_{SP,0}+Js_{L,0}=0$$ (16)

The value of *P*_*c*,*0*_, and thus of *NFD*_*0*_, appears in Eq 17 in the water flow component of the equations for *Js*_*LP*_ and *Js*_*SP*_ (Eqs 4 and 10). The optimal value of *NFD*_*0*_ was computed with a numerical procedure to solve Eq 17.

### Numerical and statistical tools

The model was entirely implemented in MatLab^®^. The main system of differential equations for *Vp*, *Vi*, *Mp* and *Mi* was solved using the classic Runge-Kutta 4,5 method (function *ode45*).

The majority of the parameters of the model were chosen a priori based on the literature, on the pre-HD values measured in the patients, or calculated from steady-state equations. The values of *α*_*LP*_ and *LpS* were left free to change for each patient, in order to obtain a better fit of the output of the model to the clinical data. The initial estimate assumed for *α*_*LP*_ and *LpS* was taken from common values reported in literature (0.05 and 5 mL/min/mmHg, respectively [10, 23]).

The fitting of said parameters was carried out with a global optimization algorithm (*particle swarm optimization*, [37]) was used to minimize the relative root mean square error (*RMSE*) of the outputs of the model relative to both clinical plasma volume and total protein concentration profiles:

$$RMSE=\sqrt{\frac{1}{N}\cdot \left[{\sum_{}^{}\left(\frac{V_{p,SIM}-V_{p,DATA}}{V_{p,DATA}}\right)}^{2}\right]}+\sqrt{\frac{1}{M}\cdot \left[{\sum_{}^{}\left(\frac{C_{p,SIM}-C_{p,DATA}}{C_{p,DATA}}\right)}^{2}\right]}$$ (17)

Where the subscripts _SIM_ and _DATA_ refer to the simulated and measured values, and *N* and *M* are the number of available measurements for plasma volume and total proteins, respectively.

Moreover, also the initial values of plasma volume and plasma total protein concentration were left free to vary during the optimization procedure, albeit only in a 10% range centered on the clinical data. This was done to obtain a better fit of the solutions of the model to the data without straying too far from the measured values, and as a way to account for measurement errors.

A third-party toolbox (*SAFE* package, [38]) was used to perform a global sensitivity analysis for a selection of parameters, using the elementary effects method [39, 40]. The sensitivity factors (elementary effects) were calculated on a model simulation of the data for an ideal patient having the average characteristics of the patients of our cohort, fixing the values of the unknown parameters to the average of the optimal values found previously, and varying only one parameter at a time.

For those fixed parameters associated with high sensitivity indices and having particular physiological relevance, the impact of the choice of their a priori value was further investigated. Specifically, each session was simulated with two different values of *r*_*SP*_, *α*_*SP*_, *LS*, and four values of *R*_*0*_ (initial interstitial-to-plasma protein concentration ratio), estimating each time a new set of optimal values of *α*_*LP*_, *LpS*, *V*_*p*,*0*_ and *C*_*p*,*0*_.

The results of the model were analyzed to assess differences and correlations between the hemodialysis sessions. The statistical analysis was carried out with STATISTICA and R. Due to the restricted number of cases at disposal, nonparametric statistics were preferred for the analyses, and the comparison of groups was carried out with Wilcoxon or Friedman test. The different number of patients’ data in each session was treated as missing cases in the Friedman test applied to homogenously sized variables. Two-way repeated measures ANOVA was used to assess the interaction effect of different HD sessions and different versions of the model (with different fixed parameters used).

## Results

### Patients characteristics

The detailed description of pre-HD fluid status and characteristics of the patients is reported in Table 3. HD1 had a longer pre-dialytic period comporting higher fluid overload. Pre-dialytic values of body weight, fluid overload, and extracellular water volume were significantly higher in HD1, compared to HD2 and HD3, in which they were similar (Table 3). No significant difference in normo-hydrated body weight (NHBW) was found; its value remained comparable before and after water removal, and between sessions, suggesting that changes in body weight were—as expected—caused by changes in fluid status. Mean arterial pressure was higher in HD1 but with only tendency to statistical significance (p = 0.075). No significant difference in sodium, potassium (both in plasma and dialysate) and in serum total proteins was found between sessions.

The results of a mixed design ANOVA test showed that further categorizing the sample by gender and presence of diabetes revealed no significant interactions with the analysis of the between-session differences. A significant main effect of a gender variable was found only for fluid overload and mean arterial pressure, with males having higher values (p < 0.05), and only a tendency for a difference was revealed for ECV (Table 5). The main effect of diabetes was significant only for intracellular volume (Table 5).

**Table 5:** Estimated marginal means and (standard error) for selected anthropometric variables in patients placed in groups according to sex and presence of diabetes, as calculated in a mixed-design ANOVA test. Body weight (BW), extracellular volume (ECV), intracellular volume (ICV), fluid overload (FO), and mean arterial pressure (MAP). * Main effect of the ‘gender’ variable significant with p < 0.05; ** close to significance with p = 0.07. ^++^ Main effect of the ‘diabetes’ variable significant with p = 0.02.

	Gender		Diabetes	
	Females	Males	nonDM	DM
*BW (Kg)*	67.8 (5.3)	70.8 (7.3)	71.4 (4.9)	67.1 (7.6)
*ECV (L)*	14.6 (0.9)**	17.4 (1.2)**	17.0 (0.8)	15.0 (1.2)
*ICV (L)*	13.5 (0.9)	15.7 (1.3)	16.3 (0.9) ^++^	12.8 (1.4) ^++^
*FO (L)*	1.8 (0.3)*	3.4 (0.4)*	2.7 (0.3)	2.6 (0.4)
*MAP (mmHg)*	87.1 (4.2)*	105.7 (5.8)*	91.6 (3.9)	101.1 (6.0)

Almost all fluid removed (HD1: 2.75L, HD2: 1.9L, HD3: 2.15L) seems to come from the extracellular space; the change in extracellular volume (ECV, HD1: 2.5L, HD2: 1.8L, HD3: 1.9L) is big compared to the change in intracellular volume (ICV, HD1: 0.35L, HD2: 0.4L, HD3: 0.1L). Note that minor discrepancies between the sum of changes in body water compartments and fluid removed occur because the latter was calculated not from bioimpedance but from changes in body weight (deemed a more accurate measure). Because of the similar fluid status between the second and the third session of the cycle, the results of the model are be shown only for HD1 and HD3.

### Parameters of the model and general behavior

**Fig 2:**
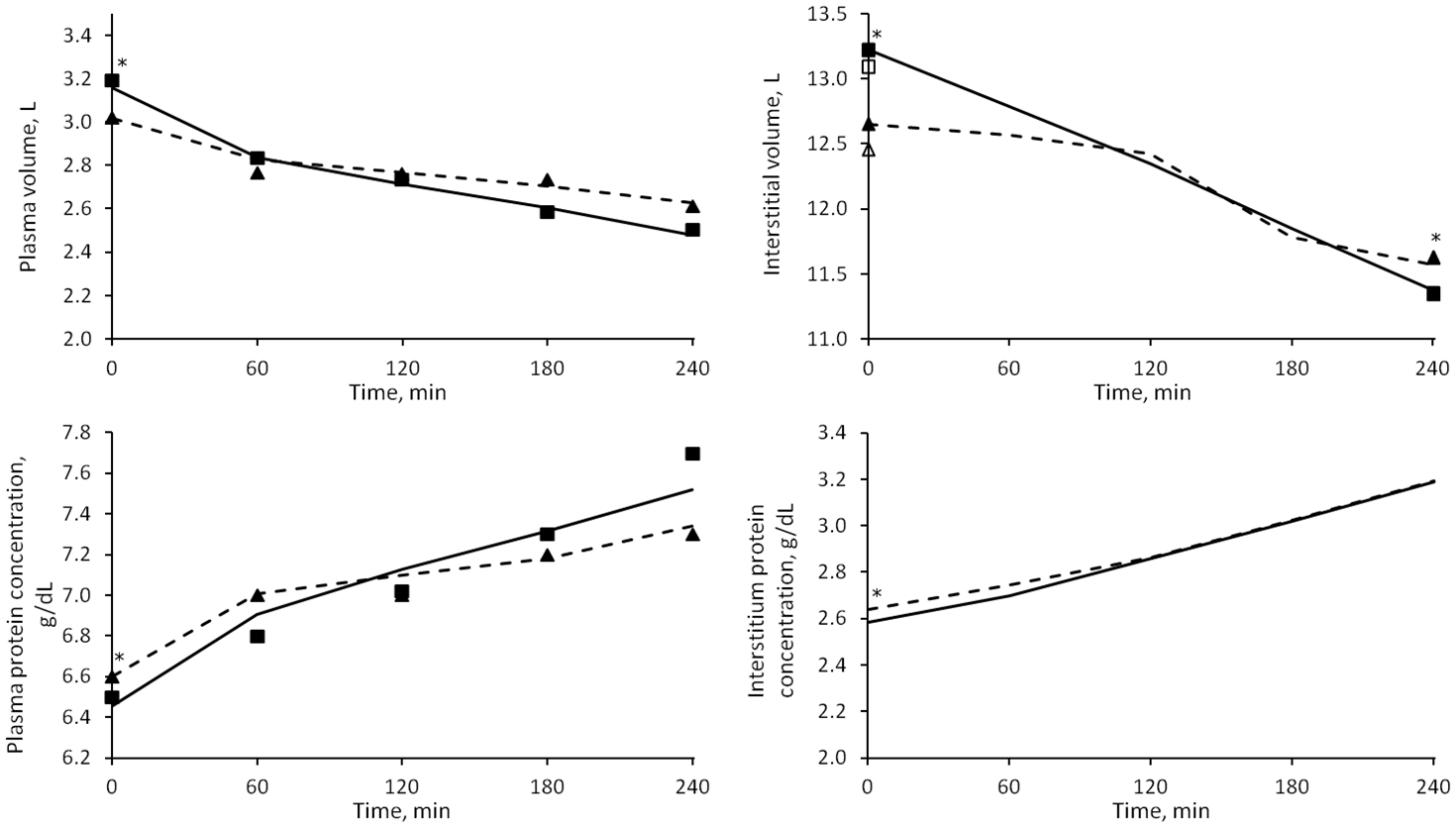
Median values of the solutions of the model (V_p_, V_i_, C_p_, C_i_). HD1 (continuous line) and HD3 (dashed line). Squares: HD1 data; triangles: HD3 data. Empty squares and triangles represent the original, unadjusted initial values of interstitial volume (see Methods section). * = p < 0.05 for HD1 vs. HD3.

The model generally fitted well the data for all patients in all three sessions (RMSE < 2%), without significant differences due to the different fluid status of the patients. The solutions of the model compared to the measured data are shown in Fig 2.

The estimated values of initial *V*_*p*_ and *C*_*p*_ were not far from the ones measured (used as starting point for the optimization); the median deviation for both is ~1%. The two unknown parameters that were estimated, *α*_*LP*_ and *LpS*, as well as other quantities calculated by the model, are reported in Table 6. Of these, the initial value of interstitial hydraulic pressure and *V*_*p*,*0*_ was found to be higher, and *C*_*p*,*0*_ lower, in HD1 (p < 0.05), while no significant difference between sessions was revealed in the others. Gender and presence of diabetes seemed not to influence the value of the parameters presented in Table 6.

**Table 6:** Comparison of the parameters of the model for sessions 1 and 3, in medians [quartiles]. The first four parameters are estimated through minimization of an error function (RMSE). V_p,0_ and C_p,0_ are estimated in a 10% neighborhood of the measured values of plasma volume and total protein concentration shown in Table 3. * p < 0.05 for HD1 vs. HD2 and HD3.

	HD1	HD3
*V*_*p*,*0*_ *(L)*	3.16 [2.82, 3.51]*	3.01 [2.53, 3.30]
*C*_*p*,*0*_ *(g/dL)*	6.45 [6.00, 6.66]*	6.60 [6.33, 6.92]
*α*_*LP*_	0.050 [0.022, 0.082]	0.045 [0.018, 0.075]
*LpS (mL/min/mmHg)*	5.82 [3.72, 9.87]	6.12 [3.77, 11.46]
*PS*_*LP*_ *(mL/min)*	0.80 [0.59, 1.08]	0.67 [0.48, 1.11]
*PS*_*SP*_ *(mL/min)*	0.09 [0.06, 0.16]	0.10 [0.06, 0.18]
*Total PS (mL/min)*	0.97 [0.76, 1.15]	0.76 [0.54, 1.17]
*Total σ*	0.939 [0.910, 0.964]	0.943 [0.916, 0.968]
*P*_*i*,*0*_ *(mmHg)*	1.10 [0.02, 2.1]*	0.78 [-0.45, 1.61]
*P*_*c*_ *(mmHg)*	17.74 [15.93, 20.03]	17.67 [15.92, 19.23]
*RMSE (%)*	1.7 [1.4, 2.4]	1.5 [1.1, 2.2]

**Fig 3:**
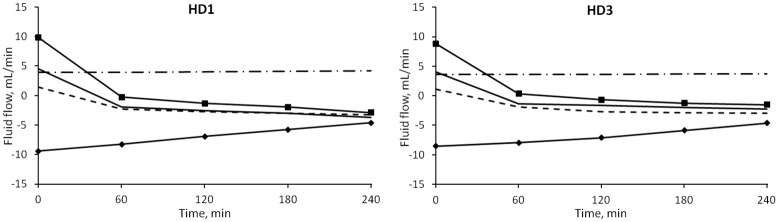
Median values of the simulated fluid flows between vascular and interstitial space during HD. Line with squares: net filtration through pores; line with diamonds: lymphatic flow; dot and dash line: large pores; continuous line: small pores; dashed line: ultrasmall pores.

The median values of the transport of fluid between plasma and interstitial compartments as calculated by the model are shown in Fig 3. In this study it was assumed that a flow with negative sign is directed toward the plasma compartment.

**Fig 4:**
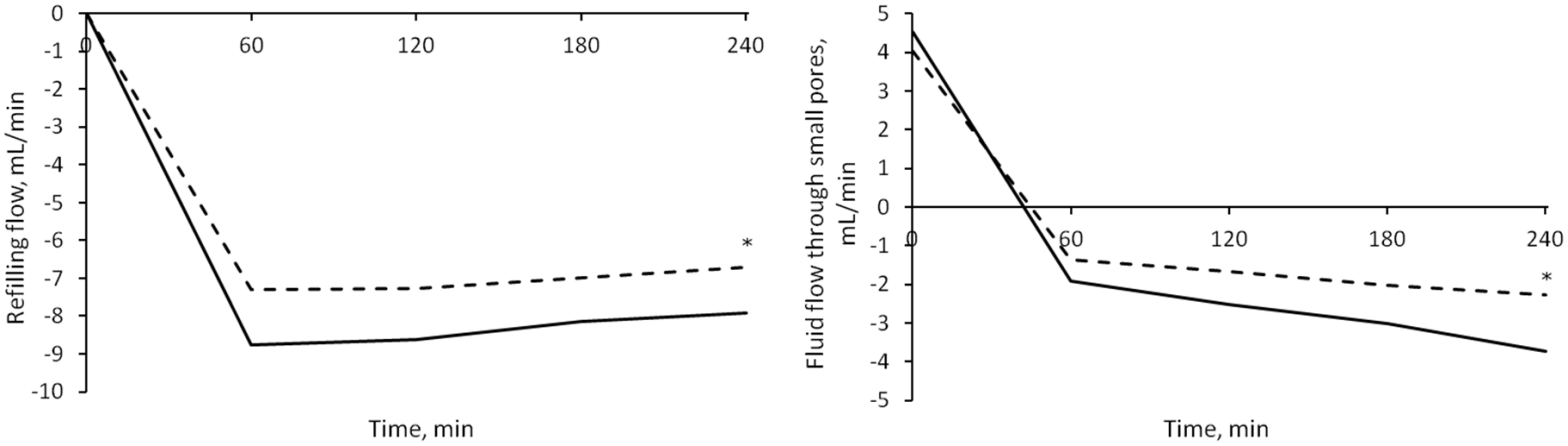
Fluid flows in different sessions. Median values of refilling flow (left panel) and small pores flow (right panel) in HD1 (continuous line) and HD3 (dashed line). The initial values were similar in both sessions, while the final values were significantly higher (in module) for HD1 (* = p < 0.0001).

Initially the flow through each of the three types of pores of the capillary membrane was directed outside of the plasma compartment; after ~1 hour the flows through small and ultrasmall pores switched direction (i.e., sign) due to the increase in oncotic pressure difference caused by hemoconcentration. At t = 0 the flows showed no significant difference between the sessions. By t = 4 hours the intensity of *Jv*_*REF*_ was significantly higher in HD1 (p = 0.0007), as were those of *Jv*_*SP*_ (and thus of the convective flow of proteins *Js*_*SP*_) and *Jv*_*UP*_, with p = 0.001 and p = 0.005 respectively (Fig 4). No interaction effect caused by gender and diabetes subcategories was found.

**Fig 5:**
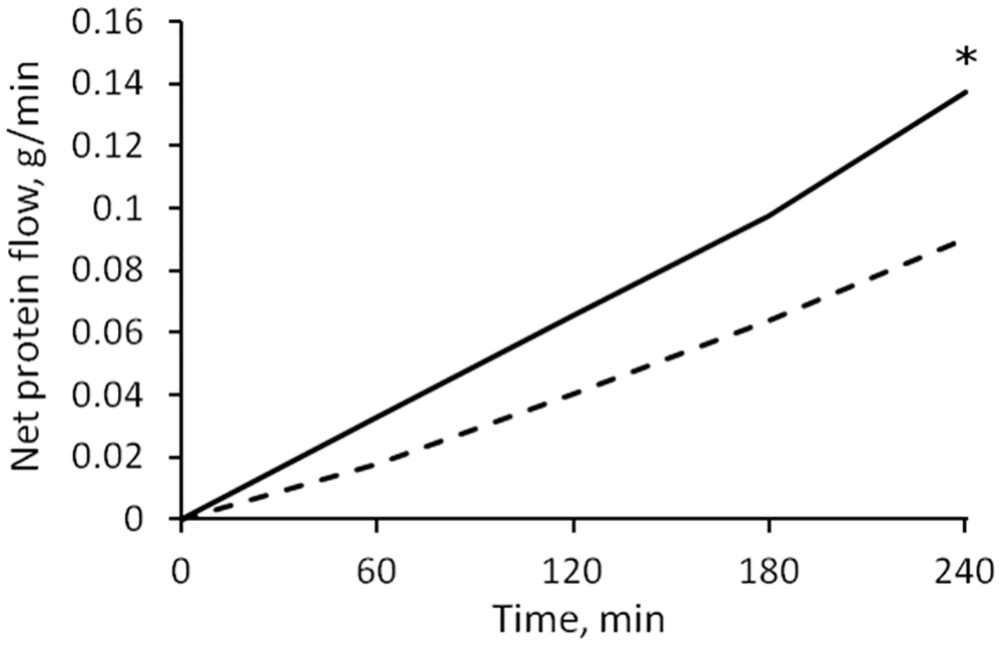
Net filtration flow of protein from vascular compartment to interstitium during water removal. At t = 240’ there was a higher escape of protein in HD1 (continuous line) than in HD3 (dashed line); * = p < 0.01.

The water removal caused a net leakage of proteins from the plasma compartment into the interstitium, because of the increase of protein concentration; the net flow of protein (difference of total transport through the capillary membrane and lymphatic reabsorption) was significantly higher for HD1 after 4 hours of treatment (Fig 5, p = 0.009), and was similar in HD2 and HD3.

**Fig 6:**
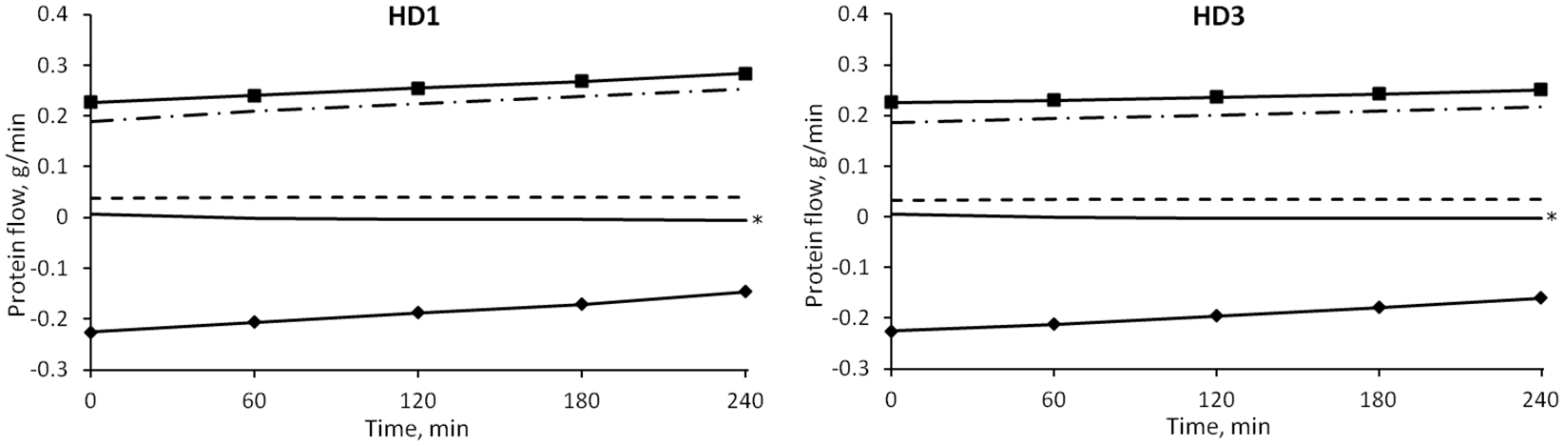
Median values of the simulated protein flows between vascular and interstitial spaces during HD. Line with squares: net flow through pores; line with diamonds: lymphatic flow; dot and dash line: convection through large pores; continuous line: convection through small pores; dashed line: total diffusion. * = p < 0.05 for HD1 vs. HD3.

The individual components of the net filtration flow of proteins according to the model are shown in Fig 6. For all the duration of water removal, the main determinant of the total protein transport across the capillary wall was the convective flow through large pores, followed by the total diffusive flow (of which diffusion through large pores constituted the 80–90%). Convective flow of protein through small pores changed from filtration to refilling after around 1 hour, but accounted for a negligible contribution (around two order of magnitude lower than large pore flow), reabsorption of protein took place almost entirely via the lymphatic system, at all times.

### Variation of the parameters

**Fig 7:**
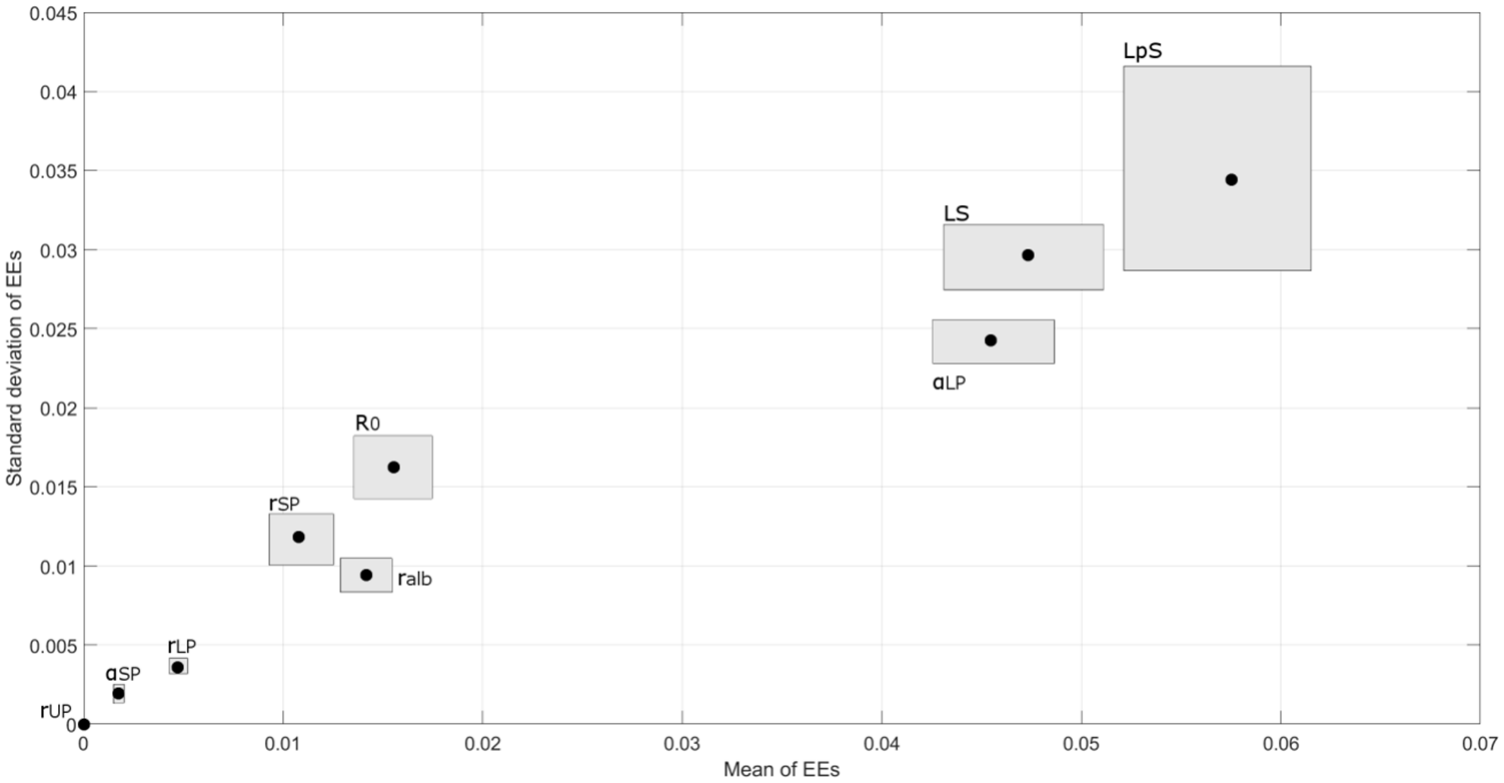
Sensitivity analysis. Sensitivity indices (circles) of some parameters of the model, calculated using the elementary effects (EEs) method. A bootstrap sample of size 200 was used to calculate the 95% confidence intervals (the area around each circle). The average of the EEs assesses the overall importance of a factor on the model output; the standard deviation is related to non-linear effects and interactions. The lower the value of both indices, the less impactful variations of the parameter are on the results of the model.

The results of global sensitivity analysis performed on some of the parameters of the model are shown in Fig 7 (for HD1 only).

The model was also implemented with different values of *r*_*SP*_, *α*_*SP*_, *LS* and *R*_*0*_ (changing one parameter at a time). Because each new version of the model was fitted to the data, the output was almost unchanged, and the change in the chosen parameter was reflected in the internal flows and other quantities calculated by the model. In each case no significant interaction was found between the effect of different values of the chosen parameter and different sessions, so the following considerations are valid for each of the three sessions (detailed numerical results thus refer to HD1).

### Fractional small pores contribution to total membrane hydraulic permeability (α_SP_)

**Fig 8:**
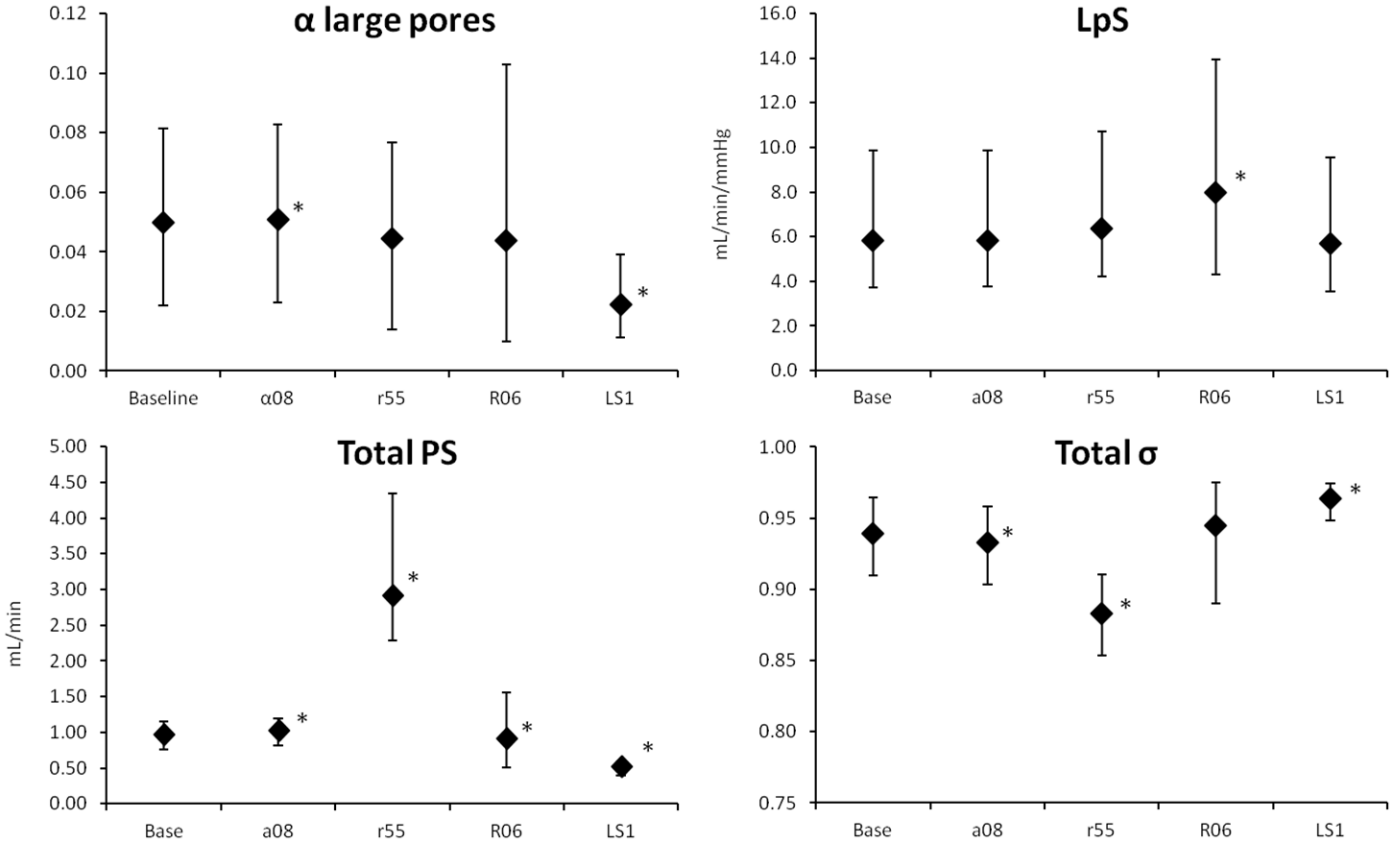
Median values and quartiles of several parameters for different versions of the model. Parameters for the baseline model were: r_SP_ = 45Å, α_SP_ = 0.6, R_0_ = 0.4, LS = 0.4. Legend: α08) α_SP_ = 0.8; r55) SP radius = 55 Å; R06) initial C_i_/C_p_ ratio = 0.6; LS1) lymphatic sensitivity = 1.0. * = p < 0.05 vs. baseline. Only HD1 data are shown.

**Fig 9:**
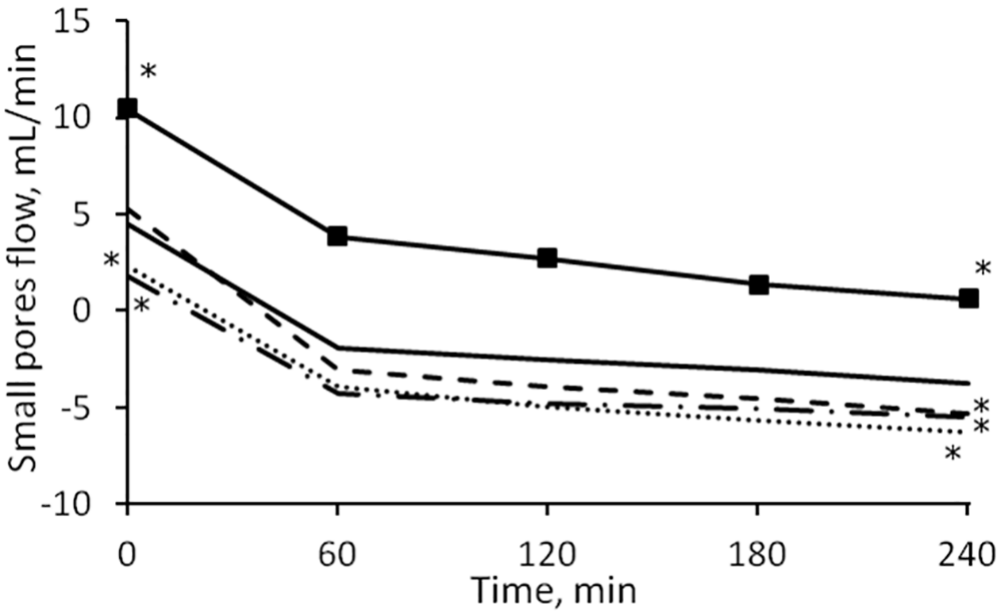
Median values of fluid flow through small pores calculated for different versions of the model. Continuous line: baseline model (r_SP_: 45Å, α_SP_: 0.6, R_0_: 0.4, LS: 0.4); line with squares: r_SP_ 55Å; dashed line: α_SP_ 0.8; dot and dash line: R_0_ 0.6; dotted line: LS = 1.0. * = p < 0.01 against baseline value (only initial and final points tested).

Since the fractional contribution to *LpS* of large pores (*α*_*LP*_) is estimated from the data, changing the value of *α*_*SP*_ also determines a variation in the contribution of ultrasmall pores (*α*_*UP*_), as for Eq 1. *α*_*SP*_ was increased from 0.6 to 0.8, therefore decreasing at the same time the maximum possible value of *α*_*UP*_. This caused changes in the estimation of α_LP_ and in almost all those parameters connected to pore transport (Fig 8); however, even if statistically significant with p < 0.05, the majority of changes were less than 2% of the original value, with the exception of an increase of ~36% in the values of *PS*_*LP*_. Thus the only relevant difference in the flows was observed in *J*_*SP*_ and *J*_*UP*_ (the former shown in Fig 9).

### Small pores radius (r_SP_)

The radius of small pores was increased from 45 to 55 Å. The increased *PS* and decreased *σ* for small pores caused a significant change in the whole-membrane values of these parameters (p < 0.0001, Fig 8). However, the estimation of the other parameters was not affected and no significant difference was found. The bigger size of small pores accounted for increased water transport (Fig 9) and an increase in both the convective and diffusive flows of proteins. The model compensated the changes in fluid and protein flow with increased lymphatic absorption.

### Initial interstitial-to-plasma total protein concentration ratio (R_0_)

**Fig 10:**
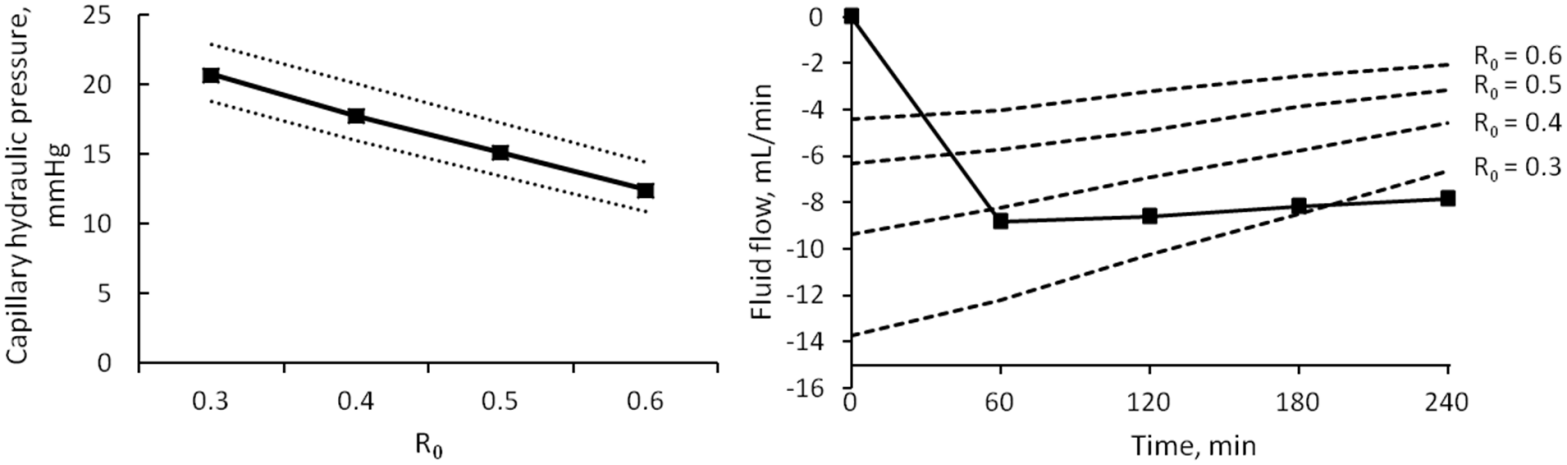
Effects of changing R_0_. Left panel) linear correlation between different values of R_0_ and calculated capillary hydraulic pressure. Median values of P_c_ (continuous line) and quartiles (dotted lines). Right panel) values of lymphatic flow obtained with different R_0_ (dashed lines), compared to the total refilling flow (line with squares).

The model was evaluated for four values of *R*_*0*_, namely 0.3, 0.4, 0.5 and 0.6. Among the estimated parameters, only the changes in *LpS* were statistically significant, with p < 0.001 (Fig 8). *PS* for both SP and LP was increasing with increasing *R*_*0*_ (p < 0.05); capillary hydraulic pressure was decreasing linearly with each increasing value of *R*_*0*_ (p ≈ 0, Fig 10, left). All flows of fluid and proteins through the capillary membrane were generally decreasing in intensity with increasing *R*_*0*_, however not linearly (with significant difference, p < 0.05, both at t = 0 and t = 240, results shown for lymphatic flow, Fig 10, right).

### Lymphatic sensitivity to interstitial fluid pressure (LS)

**Fig 11:**
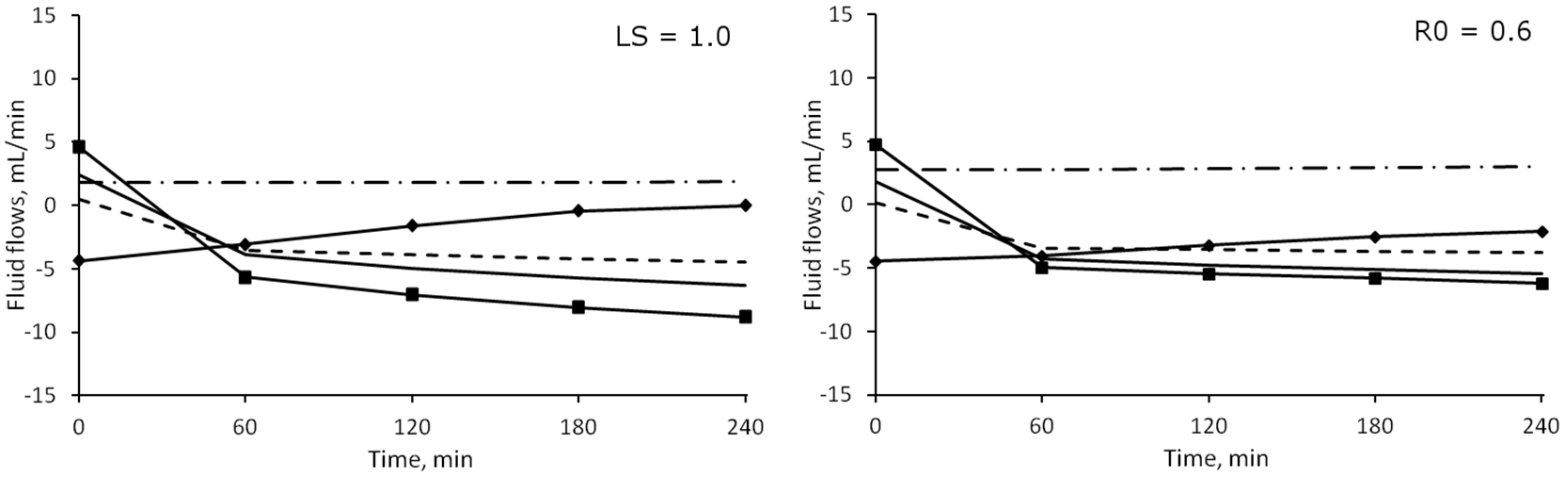
Median values of transcapillary fluid flows under different parameters. Left panel) sensitivity of lymph flow to interstitial pressure changed from 0.4 to 1.0. Right panel) initial interstitial-to-plasma protein concentration ratio (*R*_*0*_) increased from 0.4 to 0.6. Line with squares: net filtration through pores; line with diamonds: lymphatic flow; dot and dash line: large pores; continuous line: small pores; dashed line: ultrasmall pores.

The value of *LS* was increased to 1, a value between those suggested by Granger et al. for the muscle tissue (*LS* = 0.4) and skin (*LS* = 1.95), respectively [29]. The increase in sensitivity to the decrease in interstitial pressure was such that in all patients *Jv*_*L*_ reached 0 by the end of HD. The most evident effect on the parameters of the model was a marked decrease in the estimated value of *α*_*LP*_, which determined a decrease in large pores protein permeability (*PS*_*LP*_), total protein permeability (*PS*_*TOT*_) and total reflection coefficient (*σ*_*TOT*_, c.f. Fig 8). In accord with the high value of the sensitivity coefficients associated to *LS*, increasing its value determined significant changes in all solute and fluid flows, which had much lower intensity with *LS* = 1 (Fig 11a).

## Discussion

The presented model adequately described clinical data of plasma volume and serum total proteins in HD patients during each of the three HD sessions of a standard weekly treatment cycle of HD. A good fit (RMSE < 2%) was obtained using a two-compartment model of the transport of water and proteins (albumin). In order to achieve this, the model estimated a set of optimal parameters that were left free to change to account for the individual characteristics of each patient and session. This was done by minimizing an error function (Eq 12) between simulated results and data. Of the four parameters chosen for the optimization, *V*_*p*,*0*_, *C*_*p*,*0*_, *α*_*LP*_ and *LpS*, only the last two hold a special interest, as they are physiological transport parameters that are impossible to measure directly in a clinical setting but with a strong impact on the behavior of the model (see sensitivity analysis, Fig 7).

The principal difference between the first session of the week and the other two–the increased pre-HD fluid overload of the patients–was not reflected in the estimation of the parameters, with the exception of *V*_*p*,*0*_ and *C*_*p*,*0*_. The estimated characteristics of the capillary membrane showed a considerable scattering of the values both between and within patients; nevertheless, *α*_*LP*_ and *LpS* in each patient did not differ significantly from one session to the other during the one-week cycle (Table 6, p = 0.45 and 0.95 respectively), suggesting that the transport characteristics of the capillary bed do not depend on fluid overload. The values of *α*_*LP*_ and *LpS* are in good agreement with other studies that estimated (or assumed) such parameters, both in whole-body [10, 16, 17, 41] and in organ-specific situations after scaling of the results on tissue-mass [23, 27], as shown in Table 7; our estimated values are also comparable to the initial values of the refilling coefficient *Kr*, as calculated in previous studies [9, 12, 13].

**Table 7:** Values of LpS and α_LP_ from previous studies in the literature.

Source	LpS (mL/min/mmHg)	α_LP_	Notes
*Schneditz at al*., *1992* [10]	*5*.*6 ± 1*.*6*		Calculated from HD data
	*5*.*3 ± 1*.*0*		Mathematical model, estimated from HD data
*Chapple et al*., *1993* [17]	3.23		Estimated in mathematical model
*Aukland et al*., *1993* [42]	0.7–5.6*		
*Rippe et al*., *1994* [23]	5.6*	0.02–0.05	Assumed for mathematical model
*Wolf et al*., *1994* [27]	6.86*	0.139 ± 0.041	Mathematical model, estimated from animal data
*Yashiro et al*., *2002* [9]	3.76 ± 1.84		Mathematical model, estimated from HD data
*Kellen et al*., *2003* [43]		0.05	Mathematical model, estimated from animal data
*Stachowska-Pietka et al*., *2012* [44]	3.97*		Estimated in mathematical model

* To facilitate comparison, the values of LpS have been scaled to whole-body for an ideal 70 Kg patient.

The model presented is based on general physiological principles (i.e., equilibrium of Starling forces) and describes physiological processes with a mechanistic approach where possible, without exacerbating the complexity of the equations involved. However, some experimentally obtained relationships were included in the description. The relationship between interstitial fluid volume and pressure, based on the work of Ebah and colleagues [33], was obtained empirically by regressing data measured in chronic kidney disease (CKD) patients and healthy subjects; although the size of their sample was modest (~30), the relationship found was strongly significant, and particularly suitable to be used in HD modelling, as the study specifically measured interstitial pressures in oedematous CKD patients. The relationship between interstitial pressure and lymphatic flow is a simple linear function adapted from [29], and it has been already used with good results in similar studies [44, 45]. The bioimpedance spectrometry method for assessing the volume fluid compartments has typically an error of ±1 L, and discrepancies between BCM measurements and other data is well within this limit. Although not as accurate as the golden standard, this method provides important information that is difficult to acquire otherwise in clinical setting, and its use is already widespread and accepted both in clinical and research applications [46, 47].

### Fluid transport and effect of interstitial protein concentration

The model describes two types of transport between vascular bed and interstitial spaces, transport of plasma water and of albumin across the pores of a heteroporous capillary membrane and through the lymphatic system. As seen in Fig 3, before the dialysis session, the flow of water through each of the three kinds of pores was initially directed outside of the vascular compartment, the contribution of large and small pores to filtration was roughly the same, and lymphatic reabsorption was the only component of refilling flow actually directed toward the vascular space. With water removal and hemoconcentration, the increase in plasma oncotic pressure generated enough osmotic recall through small and ultrasmall pores to reverse their flow after 1 hour, Fig 3, at which time the maximum intensity of the refilling flow is reached (Fig 4). By the end of HD, the lymphatic flow was accounting for 40–45% of the total inflow toward the vascular space, while the rest was shared by small and ultrasmall pores in equal proportions.

A similar partitioning of the fluid flows was observed in different sessions (Fig 3). When altering the value of the pre-dialysis interstitial-to-plasma protein concentration ratio (*R*_*0*_), the results are however very different (Fig 11b). As already mentioned, an increase in the value of *R*_*0*_ involves a decrease in the difference between interstitial and vascular Starling forces, and thus reduced flows; the lower initial values of the flows’ intensity meant that the change of direction from filtration to refilling happened faster, with the flows of small and ultrasmall pores becoming the dominant factor for refilling over lymphatic absorption after 1 hour (Fig 11b).

The values of initial whole-body lymphatic absorption calculated with the baseline model (*R*_*0*_ = 0.4) lie well outside of the normal range reported by other physiologists to be 1–3 mL/min [48] or estimated with similar compartmental models [16, 17]. However, none of those studies were performed on hemodialysis patients, and it is possible that the chronic state of fluid overload generates a different, non-physiological equilibrium with heightened intensity of filtration and reabsorption. Moreover, after scaling the values on 100 g of soft tissue (here assumed to account for 85% of body weight to account for bone structure), our value of initial net filtration (and thus *J*_*L*,*0*_, 18.96 μL·min^-1^·100 g^-1^) and LpS (11.3 μL·min^-1^·mmHg^-1^·100 g^-1^) are similar to those reported in [23] applying a two-pore model to data of albumin transport in dog paws [49], yielding a *LpS* value of 9.05 μL·min^-1^·mmHg^-1^·100 g^-1^ with relative *J*_*L*,*0*_ of 18.5 μL·min^-1^·100 g^-1^ (although in this case the characteristics of the pores were estimated as well and were different from the present study).

The results obtained with *R*_*0*_ = 0.6 are more in line with the physiological lymphatic flows measured in both human and animal subjects [17, 42]; it is interesting to note, however, how the variations in the equilibrium of the Starling forces introduced in the model by different values of *R*_*0*_ are balanced by linear changes in *P*_*c*,*0*_ (Fig 9), which guaranteed that the output kept close to the data even with the modified parameter. The value of *P*_*c*,*0*_ ~ 17–20 mmHg, obtained with a baseline *R*_*0*_ = 0.4 or lower, is in good accordance with the average capillary pressure measured in classic studies by Guyton [35, 36]; however in later works it was concluded that such high values of *P*_*c*,*0*_ were probably due to measurements being carried out on fluid overloaded tissue, and a lower capillary pressure of 10–12 mmHg was measured [50–52], similar to what estimated in other physiological models [16, 17] and by our own model for *R*_*0*_ = 0.6.

The value of *R*_*0*_ itself is still open to debate, with some authors suggesting it should be 0.3 [10, 53], rather than close to 0.5 [42] or even higher [54]; observation of the value of *P*_*c*,*0*_ estimated by our model in relation to the assumed *R*_*0*_ supports the evidence in favour of a higher ratio of interstitial-to-plasma protein concentration.

### Effect of lymphatic sensitivity parameter

Lymphatic reabsorption plays a major role in the preservation of homeostasis, providing a continuous refilling flow whether absorption through the capillaries takes place typically after an hour (Fig 3). Even though its estimated value changes depending on the other parameters of the model, lymphatic flow is important compared to capillary refilling during the whole HD session. The value of lymphatic sensitivity to pressure changes (*LS*, Eq 5) was initially proposed for muscle tissue samples, and it is here used on the assumption that the main part of lymphatic transport occurs in muscles. The sensitivity analysis performed with the *SAFE* toolbox showed that changes in this parameter are highly influential on the behaviour of the model (Fig 7). When a higher value of *LS* = 1 was used, all of the fluid flows and many transport parameters were shown to be significantly different from the baseline model (Figs 8 and 11a). The steeper decrease in lymphatic flow and the low value of *α*_*LP*_ estimated by this new implementation resulted in values of lymphatic absorption and capillary filtration that were more in line with what was reported by other authors [16, 42], and possibly closer to the physiological reality. These results suggest that the choice of this parameter should be done with care and that the use of a single whole-body value for the sensitivity parameter may be an oversimplification.

### Albumin transport

**Fig 12:**
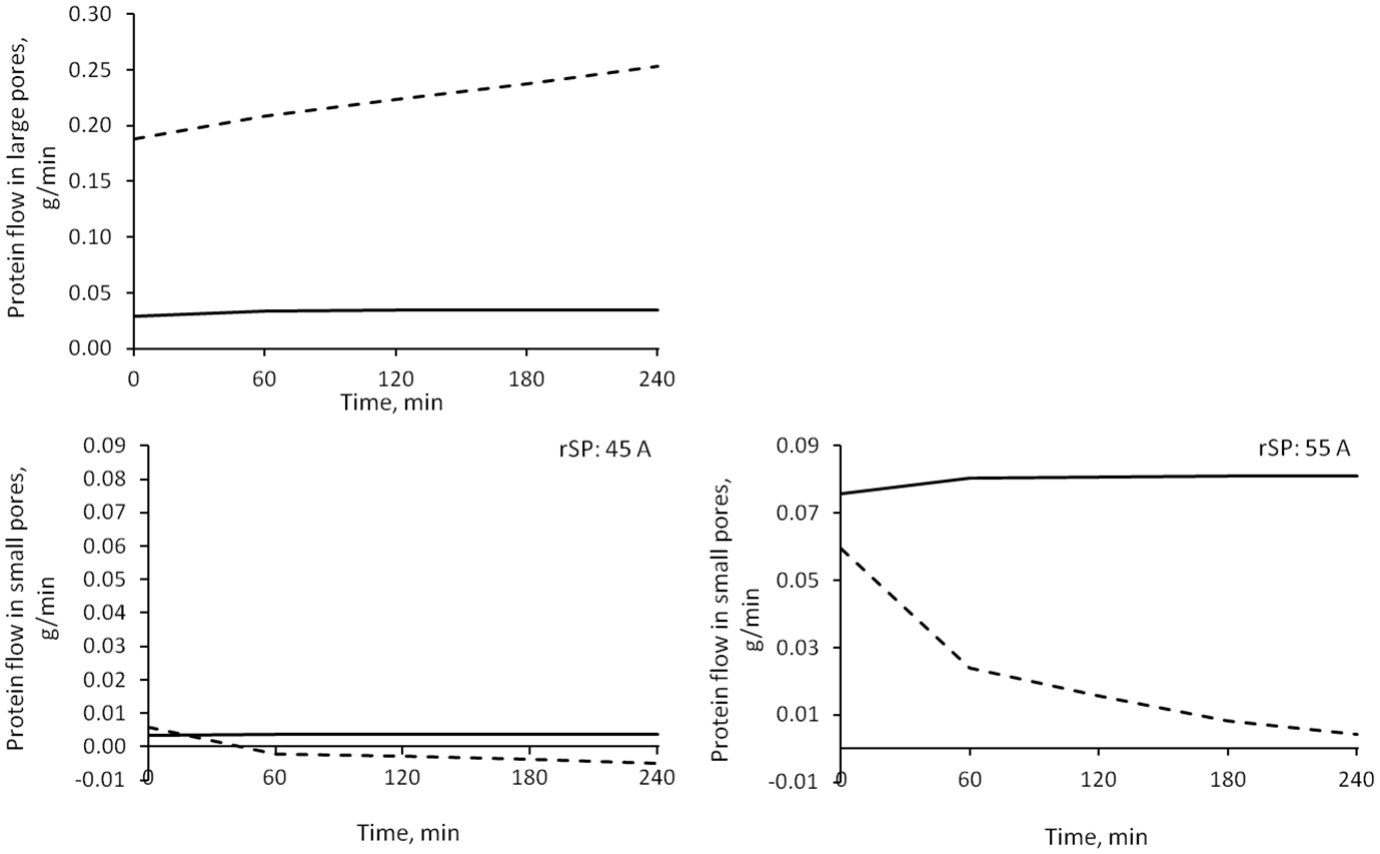
Diffusive and convective protein transport. Diffusive flow (continuous line) and convective flow (dashed line) through large pores (top panel), small pores with radius 45 Å, and small pores with radius 55 Å (bottom panels). Note the different scale of the vertical axes in the upper graph.

**Fig 13:**
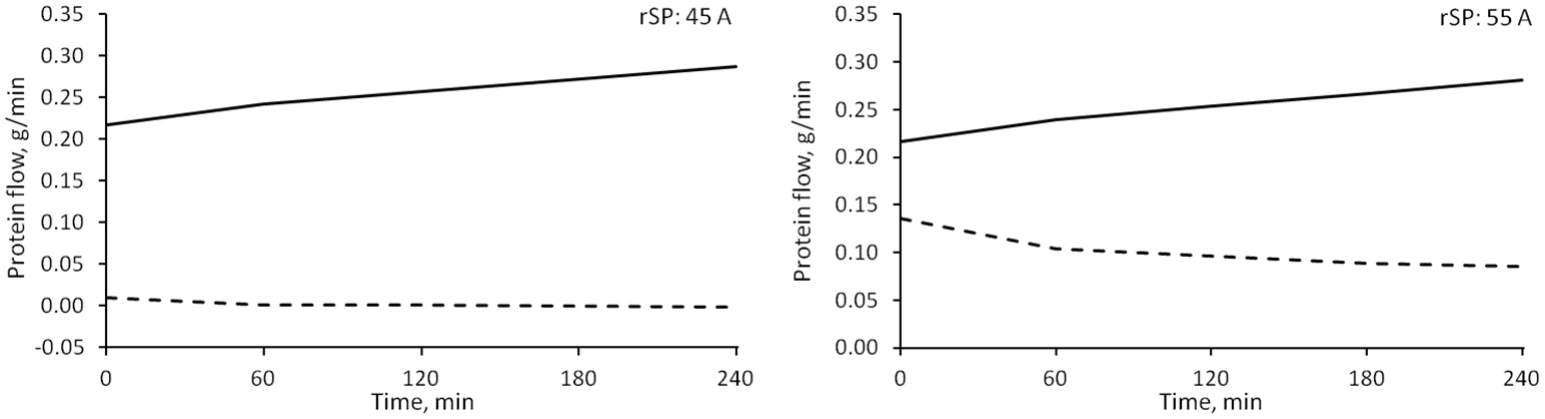
Protein transport with different small pores radii. Total albumin flow across large pores (continuous line) and small pores (dashed line). Left panel) small pores radius 45 Å. Right panel) small pores radius 55 Å.

Albumin transport across the capillary wall took place mainly via convection through the large pores (Fig 6). All types of transport, both convective and diffusive, through small pores were two orders of magnitude smaller than through large pores, despite the higher number of the former in the capillary wall. According to already reported [23] values of Péclet number (*Pe*) in large pores similar to those we found (~4), the convective transport dominates diffusion by one order of magnitude (Fig 12); however, in small pores, with -1 < *Pe* < 1, convection is of similar magnitude as diffusion even after reversing the direction of the flow. Only when we increased the radius of small pores to 55 Å, diffusion was clearly prevailing over convection (Fig 12). In large pores a net escape of albumin to the interstitium was observed for the whole dialysis time, while small and ultrasmall pores switched from filtration to refilling after circa 1 hour. However, as can be seen more clearly in Fig 13, the overall transport (sum of convective and diffusive) of albumin through small pores in the baseline model was negligible compared to transport through large pores, while increasing the radius of small pores yielded an increase in their total transport to values around 50% of large pores.

The big difference in overall albumin transport seen in the baseline model between SP and LP is attributable to the small values of permeability product *PS* calculated by the model. Such values of the total *PS* of the system (*PS*_*LP*_+*PS*_*SP*_), which we obtained (Table 2) were similar to a whole-body average value of 1.22 mL/min, calculated without using a detailed description of the capillary wall [16, 17]. However, the values of *PS* for 100 g of tissue obtained in [23] for a two-pore membrane were higher (LP: 3.3, SP: 0.75 mL·min^-1^·100 g^-1^) even when compared to our scaled values (LP: 1.4, SP: 0.18 mL·min^-1^·100g^-1^); the reason for this difference might be the presence of the water-exclusive pores in our model, across which the flow of fluid is comparable to that through SP, which takes a portion of the total water flow that would be used for convective transport through small pores in a two-pore model. For the model with radius of SP 55 Å, the scaled value of *PS* for small pores was found to be 20 times bigger (3.95 mL·min^-1^·100 g^-1^) and *σ*_*SP*_ 10% smaller, causing the oncotic force across SP to be too small to generate a refilling flow, which in this case happened only through ultrasmall pores.

### Conclusions

The model we proposed can be readily applied to individual clinical data to obtain a description of how different pathways of fluid transport interact during a hemodialysis session. Although based on a widely adopted lumped parameters approach, and classic mass balance principles, the model offers a more detailed description of the interface between compartments (adopting the 3-pore convention) than most models for hemodialysis proposed recently, granting a deeper mechanistic interpretation of the flows.

We described different factors that cannot typically be measured during clinical HD sessions, and whose values are still object of discussion. The model shows the impact of modifying the value of these parameters, which, as in the case of the interstitial protein concentration ratio *R*_*0*_, can be surprisingly high. Different values of *R*_*0*_ resulted in remarkably different intensities of lymphatic absorption (among others), ranging from being of the same magnitude to completely overshadowing all other components of the total refilling flow; at the same time however, the final output of the model was not significantly modified. These observations suggest that the choice of the parameters should be undertaken with the maximum care, even in the case of those parameters (such as *R*_*0*_) whose values appear to be readily available from the literature; the risk being, as shown, that seemingly small changes in the parameter itself can comport a big difference in the physiological processes simulated.

In conclusion, the discussed model helps us to describe and delineate events during the HD session for predictive purposes, and to obtain estimations of hidden physiological parameters that are useful for comparing and classify different patients.

## Glossary

*V*: fluid volume, mL
*M*: solute mass, g
*C*: solute concentration, g/mL
*Jv*: fluid flow, mL/min
*Js*: solute flow, g/min
*LpS*: capillary filtration coefficient, mL/min/mmHg
*P*: hydraulic pressure, mmHg
Π: oncotic pressure, mmHg
*α*: fractional contribution to capillary filtration coefficient
*σ*: Staverman’s reflection coefficient
*r*: pore radius, Å
*PS*: permeability-surface product, mL/min
*SL*: lymph sensitivity, mL/min/mmHg
*NFD*: net filtration driving force, mmHg
*R*_0_: initial value of the ratio *C*_*i*_/*C*_*p*_
*LP*: large pores
*SP*: small pores
*UP*: ultrasmall pores
*X*: pore type
*P*: plasmatic
*i*: interstitial
0: pre-HD value
*REF*: refilling
*L*: lymphatic
*DIFF*: diffusive
*UF*: ultrafiltration
*T*: value for the whole pores system
